# ExoDS: a versatile exosome-based drug delivery platform to target cancer cells and cancer stem cells

**DOI:** 10.3389/fbioe.2024.1362681

**Published:** 2024-06-05

**Authors:** Swastika Paul, Shrikrishna Bhagat, Lipsa Dash, Himadri Das Mohapatra, Sarita Jena, Suresh K. Verma, Abhishek Dutta

**Affiliations:** ^1^ EXSURE Pvt Ltd., KIIT University, Bhubaneswar, Odisha, India; ^2^ Institute of Life Sciences, Bhubaneswar, India; ^3^ School of Biotechnology, KIIT Deemed-to-be-University, Bhubaneswar, Odisha, India

**Keywords:** exosomes, cancer stem cells, breast cancer, chemotoxicity, drug delivery, mature dendritic cells, doxorubicin, ExoDS

## Abstract

Chemotherapy drugs like doxorubicin (Dox) are widely used in middle-income countries around the world to treat various types of cancers, including breast cancer. Although they are toxic, they are still widely used to treat cancer. Delivering chemotherapy drugs directly to cancer cells to reduce side effects remains a challenge. Moreover, modern research gave rise to cancer stem cell theory, which implicated cancer stem cells in tumor initiation, progression, and relapse. This makes it imperative to target cancer stem cells to achieve complete remission. Our work highlights the development of an exosome-based targeted drug delivery vehicle. These exosomes were isolated from mature dendritic cells (mDCs) and encapsulated with doxorubicin (ExoDS). Our results showed that ExoDS specifically targeted breast cancer cells and breast cancer stem cells. Further analysis revealed that ExoDS did not induce any significant apoptosis in healthy mammary cells and peripheral blood mononuclear cells (PBMCs) isolated from healthy individuals and breast cancer patients. ExoDS was also found to target circulating tumor cells (CTCs) isolated from patient blood. ExoDS also showed equal efficiency compared to free doxorubicin *in vivo*. We also observed that ExoDS reduced the expression of cancer stem cell markers in murine tumor tissues. Altogether, this work provides novel insights into how mDC-derived exosomes can be used to specifically target cancer cells and cancer stem cells.

## Graphical Abstract

**Figure:**
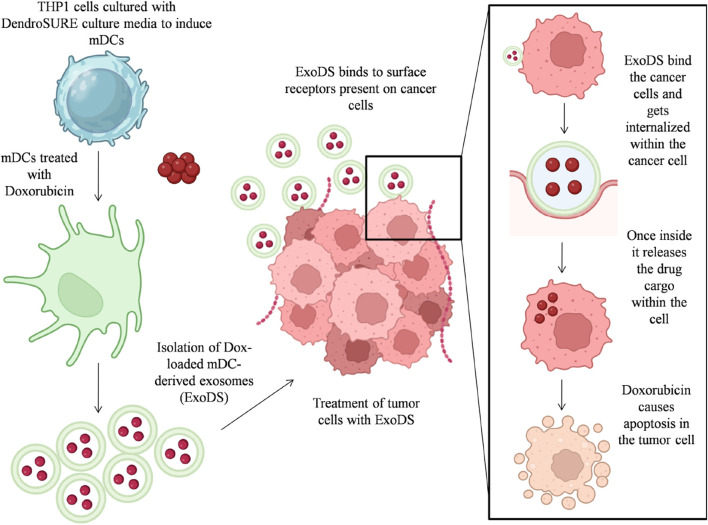
Graphical abstract showing the process workflow for preparation of ExoDS and its mode of action.

## Introduction

Doxorubicin (Dox) is a common chemotherapy drug used to treat a broad range of cancers, including breast cancer. However, significant cardiotoxicity was associated with 11% of patients exposed to doxorubicin, which limits its continued therapeutic use (Johnson-Arbor and Dubey, 2023). Another challenge which limits the use of doxorubicin is the resistance developed by cancer cells. Resistance to therapeutics can be caused by numerous factors associated with either acquired or *de novo* mechanisms, including enhanced levels of efflux (Lovitt et al., 2018; Mattioli et al., 2023).

One strategy to prevent the uptake of doxorubicin into cardiac cells and other healthy cells is the encapsulation of the drug to prevent non-specific uptake and also to improve the pharmacokinetic properties of the drug. Although encapsulated forms of doxorubicin limit the cardiotoxicity observed, they are not without their own liabilities, as an increased amount of the drug is deposited in the skin, where liposomal doxorubicin (Lipo-Dox) can cause palmar–plantar erythrodysesthesia (Schindler et al., 2019). Moreover, synthetic exogenous vehicles generate significant neurotoxicity. In addition, unmodified liposome-mediated drug delivery is limited owing to the shorter circulation time and instability (Thakur et al., 2020).

Although the packaging of the drug within delivery vehicles limits its toxicity, it remains a challenge to target these drugs specifically to drug-resistant cells. Despite significant advances in targeted therapies, cancer patients suffer from relapse due to drug resistance and the persistence of cancer stem cells (CSCs) in the tumor tissue. Although conventional therapies are effective in eliminating bulk tumor cells, the small population of CSCs left behind undergoes asymmetric division to form new stem cells as well as differentiated cells that repopulate the tumor tissue, causing relapse. More recently, cell therapy emerged as a forerunner in targeted therapy, but almost 43.3% of patients experienced failure (Di Blasi et al., 2022). The reason for this high failure rate is mainly due to the heterogeneity of the tumor microenvironment and tumor immune escape, which remodulates the bioengineered immune cell therapy into pro-tumor immune cells, and hence, the tumor cells and CSCs can easily escape the immune surveillance, which leads to tumor progression. CSCs, being the key players in the tumor microenvironment, can strategically shed some factors or suppressive signals that suppress the immunogenicity of the cell therapy products and lead to the failure of cell therapy in the long run. Hence, the need of the hour is to eliminate the CSCs from the root of the tumor tissue.

Dendritic cells (DCs) are the sentinels of the immune system that play a critical role in priming anti-tumor immunity. TNF-, FasL-, and TRAIL-induced tumoricidal function of DCs is activated as these cells arrive in the tumor beds. DCs are also known to induce tumor cell apoptosis via the caspase-8-dependent pathway. However, this anti-tumor function of DCs can be impeded by suppressive signals present in the tumor microenvironment (Del Prete et al., 2023). Therefore, there is a need to harvest this intrinsic tumoricidal property of DCs to develop novel strategies to target cancer cells and CSCs. Dendritic cells are professional antigen-presenting cells and are widely used to re-educate other immune cells to target cancer cells. DCs can mobilize multiple arms of the anti-tumor immune response. They can engulf tumor cells killed by natural killer (NK) cells and then present tumor-associated antigens to cytotoxic T lymphocytes (CTLs). DCs may also kill cancer cells by generating signals expressed at the surface of dying tumor cells, such as calreticulin, HMGB1, uric acid, or HSP. These signals may enhance DC maturation, cytokine production, and the ability to induce T-lymphocyte activation. In addition to the ability of DCs to activate CD4^+^ and CD8^+^ T cells and cross-talk with NK cells, a novel, direct tumoricidal activity of DCs has recently been described by various groups. Human blood conventional dendritic cells (cDCs) acquire the ability to kill tumor cells via TRAIL, FasL expression, or TNF-α, perforin, and granzyme secretion. Interestingly, the cytotoxicity may be a function of DC maturation since LPS or IFN-γ enhances the cytotoxicity of DCs. Nanocarriers serve as an attractive alternative for targeted drug delivery to tumors due to their size and ability to penetrate dense tumor tissue (Ertas et al., 2021; Sen et al., 2023). Exosomes are nano-sized extracellular vesicles with a size range of 30–200 nm. They are known to transfer various peptides, fatty acids, and nucleic acids, including microRNA (miRNA), to neighboring cells as cargo shuttles responsible for intercellular communication (Zhang et al., 2019). Exosomes, being naturally derived, have very low immunogenicity compared to liposomes or any other synthetic nanocarriers and also do not induce any side effects in patients (Zou et al., 2023). Moreover, unlike immune cells, immune cell-derived exosomes cannot be impeded by tumor cells. In the current study, we explore the possibility of using DC-derived exosomes as a drug carrier to specifically target cancer cells and CSCs. We developed mature dendritic cell-derived exosomes (mDC-derived exosomes) and infused them with doxorubicin to ensure the uptake of doxorubicin, specifically by breast cancer cells and cancer stem cells. These drug-loaded mDC-derived exosomes (ExoDS) also targeted circulating tumor cells (CTCs) isolated from patient blood but spared healthy cells such as peripheral blood mononuclear cells (PBMCs) and normal mammary cells. ExoDS has dual advantages, i.e., on one hand, it reduces toxicity associated with doxorubicin and precisely delivers it to tumor cells, thus reducing non-specific targeting, and on the other hand, it transports the tumoricidal properties of DCs directly to the cancer cells and CSCs, accentuating the tumoricidal potential of ExoDS. The effect of ExoDS was tested both *in vitro* and *in vivo*. Overall, our results indicated that the proposed ExoDS could be adopted as an efficient and safe long-term treatment for breast cancer.

## Materials and methods

### Generating mature dendritic cells from monocytes

mDCs were obtained from DendroSURE™ Cell Culture Media (ExSURE, India), which induce the conversion of monocytes into mature dendritic cells within 48 h. THP1 cells were cultured in RPMI 1640 Medium (HiMedia) supplemented with 10% fetal bovine serum (FBS), 250 µg/mL amphotericin B, and 0.2% penicillin–streptomycin (Baxter et al., 2020). The cells were harvested by centrifugation at 800 rpm for 5 min, added to DendroSURE™ media, and cultured for 48 h to generate mDCs.

### Loading of doxorubicin within the mDC-derived exosomes

To package doxorubicin within the mDC-derived exosome, we cultured mDCs from DendroSURE™ media in the presence of doxorubicin (1.8 µg/mL) for 24 hrs. This led to the endogenous packaging of doxorubicin within the mDC-derived exosome. These doxorubicin-loaded mDC-derived exosomes are hereafter referred to as ExoDS.

### Isolation and characterization of ExoDS

Exosome isolation from mature dendritic cells (mDCexo) and doxorubicin-packed mDC-derived exosomes (ExoDS) was carried out using the exosome isolation reagent Exosure (ExSURE, India). The cell media were collected after 48 h of incubation of THP1 cells in DendroSURE™ media. After mature dendritic cell formation, mDCexo isolation was carried out. However, for ExoDS isolation, mDCs were supplemented with DendroSURE™ media and doxorubicin, and cell culture media were harvested after 24 h. The harvested media underwent centrifugation at 4,000 rpm for 30 min at 4°C, facilitating the removal of cellular debris. The resulting supernatant was carefully transferred to a fresh tube, and the appropriate volume of an exosome isolation reagent (Exosure) was added following the manufacturer’s instructions. This solution was meticulously mixed by vortexing and maintained at 4°C in an upright position for overnight incubation. The next day, the exosomes were precipitated by subjecting the mixture to centrifugation at 13,000 rpm for 1 h at 4°C. To ensure their long-term preservation, a suitable volume of 1× phosphate-buffered saline (PBS) was added to the pellet containing the exosomes, leading to their resuspension, after which they were stored at −20°C. The isolated mDCexo and ExoDS were characterized and confirmed by measuring their size, number, and expression of exosomal surface markers (Lai et al., 2022). Microscopic observation of the exosomes by transmission electron microscopy (TEM) was carried out, followed by dynamic light scattering (DLS) analysis (Malvern, United Kingdom) and nanoparticle tracking analysis (NTA) to determine the size and number of the exosomes in respective amounts of the solution. The molecular marker expression of exosomes CD9 and CD63 was checked by Western blot analysis (Huda et al., 2021).

### Culture of primary tumor and CTCs

For the breast tumor tissue and blood sample collection from the patient, we collaborated and obtained ethical committee approval from the Oncology Department at KIMS, Bhubaneswar (KIMS/SLRC/71/2022/01), and AIIMS Hospital (T/EMF/Surg.Onco/22/111). To isolate CTCs, a fresh 3-mL blood sample was obtained from breast cancer patients at KIMS and AIIMS hospitals in Bhubaneswar with applicable informed consent. PBMCs were derived from the blood sample by adding the Leucosure PMBC isolation reagent (ExSURE) or HiSep (HIMEDIA) in an equal volume, followed by density gradient centrifugation at 1,500 rpm for 45 min. The resultant buffy layer, rich in PBMCs, was carefully extracted and subjected to secondary centrifugation at 1,500 rpm for 10 min after adding an equal volume of 1× PBS. Subsequently, the isolated PBMCs were cultured in RPMI 1640 Medium supplemented with 15% FBS (Gibco), 0.2% penicillin–streptomycin, amphotericin B (250 μg/mL), insulin (9.5–11.5 mg/mL), epidermal growth factor (EGF), B27, and hydrocortisone (1,000 μg/mL) at 37°C with 5% CO_2_. After an incubation period of 4 days, we observed the attachment of CTCs to the flask surface in 4 of the 13 samples collected from breast cancer patients (Koch et al., 2020).

### Culture of primary tumor cells

Breast tumor tissue and the corresponding normal tissue specimens were procured from KIMS, Bhubaneswar (KIMS/SLRC/71/2022/01), and AIIMS Hospital (T/EMF/Surg.Onco/22/111) from a consenting patient. The tumor tissue was meticulously washed with 1× PBS and subsequently fragmented into minute segments using a sterile scalpel. To obtain single-cell suspension, the tumor tissue fragments were subjected to enzymatic dissociation with collagenase (1 mg/mL, prepared in serum-free RPMI 1640 Medium) overnight at 37°C in an orbital shaker at 80 rpm. After enzymatic digestion, the resultant tumor tissue suspension was passed through a nylon mesh. The single-cell suspension was centrifuged at 3,000 rpm for 5 min, and the cell pellet was thoroughly washed with 1× PBS to ensure the removal of unwanted debris and substances. The isolated cancer cells were then cultured in RPMI 1640 Medium supplemented with 15% FBS (Gibco), 0.2% penicillin–streptomycin, amphotericin B (250 µg/mL), insulin (9.5–11.5 mg/mL), EGF, B27 supplement, and hydrocortisone (1,000 µg/mL). The cells were cultured for 6–7 days with specific medium changes within a controlled environment, observed regularly for tumor cells, and utilized for further study (Chen et al., 2023).

### Mammosphere formation assay

To obtain CSCs from breast cancer cell lines, MDA-MB-231, MDA-MB-468, and MCF7 were grown as monolayers, trypsinized, and seeded into ultralow-attachment plates (Corning Inc., NY, United States) at a density of 2.5 × 10^4^ cells per well. We cultured these cells in ultralow attachment in serum-free RPMI 1640 Medium (HiMedia, India), supplemented with insulin (Sigma), EGF (BioLegend), and B27 (Sigma). The cells were cultured for 4–5 days for mammosphere formation (Kai et al., 2015). The primary spheres were subjected to secondary-sphere formation by dislodging the spheres using trypsin, followed by reseeding the single-cell suspension in mammosphere-specific media at 2.5 × 10^4^ cells per well into ultralow-attachment plates. To characterize the CSCs, we conducted CD44/CD24 analysis. Our analysis showed that >85% of the cells are CD44^+/^CD24^–^. We also conducted flow cytometric analysis of Nanog, Sox2, Oct4, ALDH1A1, ABCG2, and MRP1. The data are given in Supplementary Material.

### Apoptosis assay

To understand the effect of ExoDS in inducing apoptosis, we cultured breast cancer cells (MDA-MB-231, MDA-MB-468, and MCF7) and cancer stem cells derived from the mentioned cell lines and treated them with ExoDS and free Dox. The percentage of Annexin V-positive cells was determined by flow cytometry (Kabakov and Gabai, 2018).

### Flow cytometric analysis

After the complete treatment of THP1 cells with DendroSURE media, the cells were harvested, washed, and dissolved in 50 µl of 1× PBS. For the detection of differentiation and maturation in DCs, the cells were stained with fluorescent dye-conjugated mAb against selected markers (anti-CD14, anti-CD40, anti-CD83, and anti-CD86) (Beckman Coulter). The percentage of positive cells was evaluated using the CytoFLEX LX Flow Cytometer (Beckman Coulter, United States), and data were analyzed using CytExpert software (Lieschke et al., 2022).

### Fluorescent labeling of ExoDS

For tagging ExoDS with CFSE dye, we labeled THP1 cells with the CFSE dye as per the instructions given (CellTrace™ CFSE Cell Proliferation Kit). Following the tagging of THP1 cells, the tagged cells were seeded in DendroSURE Cell Culture media for 48 h. Following incubation, the conditioned media were harvested, and the labeled exosomes were isolated using the Exosure exosome isolation reagent.

For tagging ExoDS with 1′-dioctadecyl-3, 3, 3′, 3′-tetramethylindotricarbocyanine iodide (DiR) dye, the purified exosomes were labeled with the fluorescent dye, DiR, DilC18 (Invitrogen, United States and Canada, D-12731). The exosomes were incubated with DiR for 30 min at 37°C in the dark. After incubation, the exosome isolation reagent was added and incubated at 4°C overnight for labeled exosome isolation. After centrifugation at 13,000 x g for 1 h, the green pellets of the labeled exosomes were re-suspended in 1× PBS. The labeling of exosomes was tested at an excitation wavelength of 754 nm and an emission wavelength of 778 nm using the IVIS Spectrum Imaging System, and the exosomes were tracked after injection in mice at a specific interval of time (Santelices et al., 2022).

### Immunofluorescence-based detection of CD9 and CD63

The cells were seeded on 12-mm-diameter coverslips. Following the differentiation of THP1 cells to mDCs after 48 h, the cells were fixed with 4% paraformaldehyde (PFA) (EMS) for 15 min at room temperature (RT). Primary and secondary antibodies were successively incubated for 1 h each at RT in PBS containing 0.2% BSA. The cells were permeabilized in 0.3% Triton X-100 + 5% BSA (in PBS) for 15 min at RT and blocked in 5% BSA (in PBS) for 30 min at RT; the primary antibody was incubated overnight at 4 °C, and the secondary antibody was incubated for 1 h at RT, both in blocking solution. Coverslips were then mounted on slides with DAPI (Invitrogen). Images were acquired on a Zeiss LSM 780 Confocal Microscope using a ×63 objective. At least 5 fields were captured to image a total of at least 10 cells per experiment (2 independent experiments). Image analysis was performed using ImageJ.

### *In vivo* efficacy test of ExoDS

To study the toxicity of ExoDS on NOD/SCID mice (females), all the animals were purchased and housed in an animal house at the Institute of Life Sciences, Bhubaneshwar, India. The Institutional Animal Ethical Committee (Institute of Life Sciences, Bhubaneswar, India) approved the use of animals (Project no: ILS/IAEC-308-AH/NOV-22). All the methods associated with animal studies were performed according to the guidelines of the Committee for the Purpose of Control and Supervision of Experiments on Animals (CPCSEA), India. MDA-MB-231 cells (1 × 10^6^) re-suspended in GFR Matrigel (Corning) were injected subcutaneously into the left mammary flanks of 6-week-old female NOD/SCID mice (n = 11). A total of eight (72.72%) of the injected mice developed subcutaneous tumors of palpable size by day 7 after tumor cell injection. For the toxicity study experiment, tumor-bearing mice were randomly assigned to four treatment groups (n = 2 in each group for a dose–response experiment): a) control exosome in PBS; b) ExoDS; c) free doxorubicin drug; and d) saline. The treatment was given every 48 h s for the next 12 days (6 dosages). The tumor volume and weight of the mice were calculated before every dose was administered. All the mice were sacrificed after 48 h of final treatment, and the subcutaneous tumors were excised along with the major vital organs (lung, kidney, heart, spleen, and liver) and normal tissue adjacent to the tumor site and stored in 10% formalin for further use (Warin et al., 2010).

The tumor volume of the mice was calculated using the following formula:

$$\text{Tumor Volume}=\text{Length of Tumor }x\;{\left(\text{Width}\;\text{of}\;\text{Tumor}\right)}^{2}/2.$$

### Western blot analysis

The cell suspension of MDA-MB-231 cells and CSCs obtained from it and tumor tissues was suspended in RIPA buffer (20 mM Tris-HCl, pH 7.5, 150 mM NaCl, 1 mM Na_2_ EDTA, 1 mM EGTA, 1% NP-40, 1% sodium deoxycholate, 2.5 mM sodium pyrophosphate, 1 mM β-glycerophosphate, and 1 mM Na_3_VO_4_) containing the protease inhibitor, followed by sonication (amplitude 35%, 5 s on and off for 10 min) and centrifugation (12,000 rpm, 15 min at 4°C). The protein was isolated and quantified using the Bradford assay. Equal amounts of protein were heated at 95°C for 10 min, resolved by 10% SDS-PAGE, and transferred onto polyvinylidene difluoride (PVDF) membranes (Merck). Then, the membranes were blocked with 5% BSA in 1× Tris-buffer saline with Tween 20 (TBST) and probed with specific primary antibodies overnight. The secondary HRP-conjugated goat anti-rabbit IgG antibody (ABclonal; 1:5,000) was used according to the manufacturer’s protocol. Antibody binding was detected using the chemiluminescence substrate and the ImageQuant LAS 500 System. The antibodies used included GAPDH (ABclonal; 1:2,000), beta-actin (ABclonal; 1:2,000), caspase-8 (ABclonal; 1: 1,000), CD63 (ABclonal; 1: 1,000), CD9 (ABclonal; 1:1,000), LC3A/LC3B (ABclonal; 1:1,000), and secondary antibody HRP-conjugated anti-rabbit IgG (ABclonal; 1:5,000) (Mishra et al., 2017).

### Real-time polymerase chain reaction

The stemness marker gene (SOX2, OCT4, and NANOG) expression analysis was performed by quantitative real-time polymerase chain reaction (qRT-PCR). Total RNA was isolated from the MDA-MB-231 cells and tumor and normal tissue samples of mice using the TRIzol reagent (SRL, India) and quantified using a NanoDrop system. RNA was reverse-transcribed using the 5× PrimeScript RT Master Mix (Takara, Japan) in a total volume of 20 μL, and the resulting complementary DNA (cDNA) was used as a template for qRT-PCR (QuantStudio 5; Thermo Fisher). All reactions were carried out in triplicate using TB Green™ Premix Ex Taq™ II (Takara, Japan), and melt curve analysis was performed at the end of the PCR step (Iwahara et al., 2006). The Ct values of samples were normalized with the housekeeping gene *GAPDH*, and the fold change in transcript levels was estimated using the ΔΔCt method.

Primers used for the study are listed in the table.

**Table:** 

Gene name	Primer sequence
GAPDH	Fp 5ʹ-CCTGCACCACCAACTGCTTA-3ʹ Rp 5ʹ-GGCCATCCACAGTCTTCTGGG-3ʹ
NANOG	Fp 5ʹ-AGTCCCAAAGGCAAACAACCCACTTC-3ʹ Rp 5ʹ-TGCTGGAGGCTGAGGTATTTCTGTCTC-3ʹ
OCT4	FP 5ʹ-GGGCTCTCCCATGCATTCAAAC-3ʹ Rp 5ʹ-CACCTTCCCTCCAACCAGTTGC-3ʹ
SOX2	Fp 5ʹ-CCATGCAGGTTGACACCGTTG-3ʹ Rp-5ʹ-TCGGCAGACTGATTCAAATAATACAG-3ʹ

### Proliferation assay

The effect of ExoDS on the proliferation of MDA-MB-231 cells and MDA-MB-231 cell-derived CSCs was analyzed by flow cytometry. The cells were treated with four different sets: ExoDs, control exo, doxorubicin (5 ng/mL), and 1× PBS for 48 h. After incubation, the cells were harvested, washed, and dissolved in 50 µl of 1× PBS and stained with the Ki-67 proliferation marker antibody (BioLegend) for 30 min on ice (Yerushalmi et al., 2010). The percentage of proliferation was analyzed using the CytoFLEX LX System (Beckman Coulter, United States), and the proliferation index was calculated.

### MTT assay

To carefully study the cytotoxicity of ExoDS and compare it with that of free doxorubicin and liposomal doxorubicin, we conducted an MTT assay on MDA-MB-231 cells. The viability of MDA-MB-231 cells treated with ExoDS, liposomal doxorubicin, and free doxorubicin was assessed after 48-h intervals using the MTT {3-(4,5-dimethylthiazol-2yl)−2,5-diphenyltetrazolium bromide} assay. MDA-MB-231 cells were initially seeded in triplicate into 96-well plates at 5 × 10³ cells/well in 200 µL RPMI 1640 Media and cultured to confluence over 1 day at 37°C and 5% CO_2_. Upon reaching confluence, the cells were exposed to varying concentrations (0–500 ng/mL) of the aforementioned treatments. A 0.5 mg/mL MTT solution was prepared in 1× PBS, and 100 µL was added to each well after treatment, followed by 3-h incubation. After removing the supernatant, dimethyl sulfoxide (DMSO) was added to stop formazan formation. The 96-well plate was then shaken for 15 min in the dark at RT, and absorbance was measured at 570 nm (Maleki et al., 2020).

### Statistical analysis

Values are shown as the standard error of the mean unless otherwise indicated. Data were analyzed, and the appropriate significance (*p* < 0.05) of the differences between mean values was determined using a two-tailed unpaired Student’s *t*-test in GraphPad Prism 7. Data are represented as the mean ± SEM.

## Results

### Generation of mature dendritic cells from THP1 using DendroSURE™ Cell Culture Media

**FIGURE 1:**
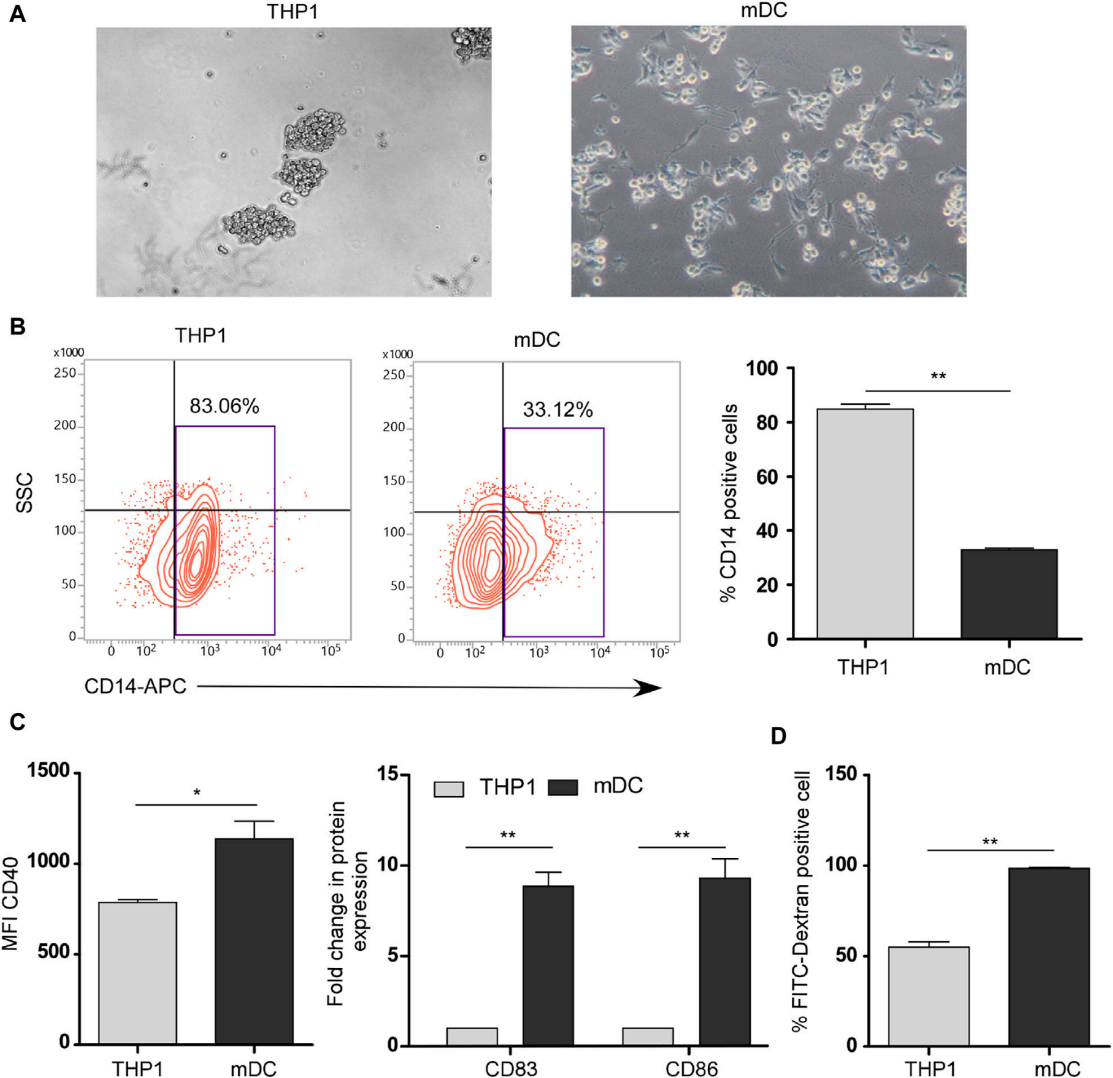
**(A)** Image analysis showing differentiation of THP1 cells to mDCs in the presence of DendroSURE Culture Media within just 48 h. **(B)** Left panel: flow cytometric analysis of CD14 expression (monocyte marker) of THP1 cells and induced mDCs. Right panel: graphical representation of the expression pattern of CD14 (n = 3). **(C)** Left panel: bar graph showing the mean fluorescence intensity (MFI) of the CD40 marker in THP1 cells and induced mDCs. Right panel: flow cytometric analysis of CD83 and CD86 (maturation markers for DCs) was conducted. Data were pooled from three independent experiments and represented in graphical format. **(D)** Bar graph showing the higher phagocytic ability of DCs than that of THP1 cells. Flow cytometric analysis was used to analyze the uptake of FITC-dextran by THP1 cells and mDCs. Data were pooled from three independent experiments. The error bar indicates SEM; * indicates *p* < 0.05, ** indicates *p* < 0.01, *** and indicates *p* < 0.001.

To induce the maturation of THP1 cells into mDCs, we cultured THP1 cells with DendroSURE™ Cell Culture Media for 48 h. Our results showed that DendroSURE™ Cell Culture Media induced the differentiation of THP1 cells to mDCs within just 48 h (Figure 1A). We next analyzed the molecular signature of the induced mDCs. First, we observed a decrease in CD14 expression (a marker for monocytes) as monocytes differentiated to mDCs (Figure 1B). Next, flow cytometric analysis confirmed significantly enhanced expression of mDC markers like CD40, CD83, and CD86 (Figure 1C). To observe whether the mDCs generated are functional, we also carried out a FITC-dextran assay to observe the phagocytic capabilities of the generated mDCs (Figure 1D). Collectively, our results confirmed the generation of mature dendritic cells from monocytes within just 48 h of culture.

### Loading of doxorubicin within mDC-derived exosomes

**FIGURE 2:**
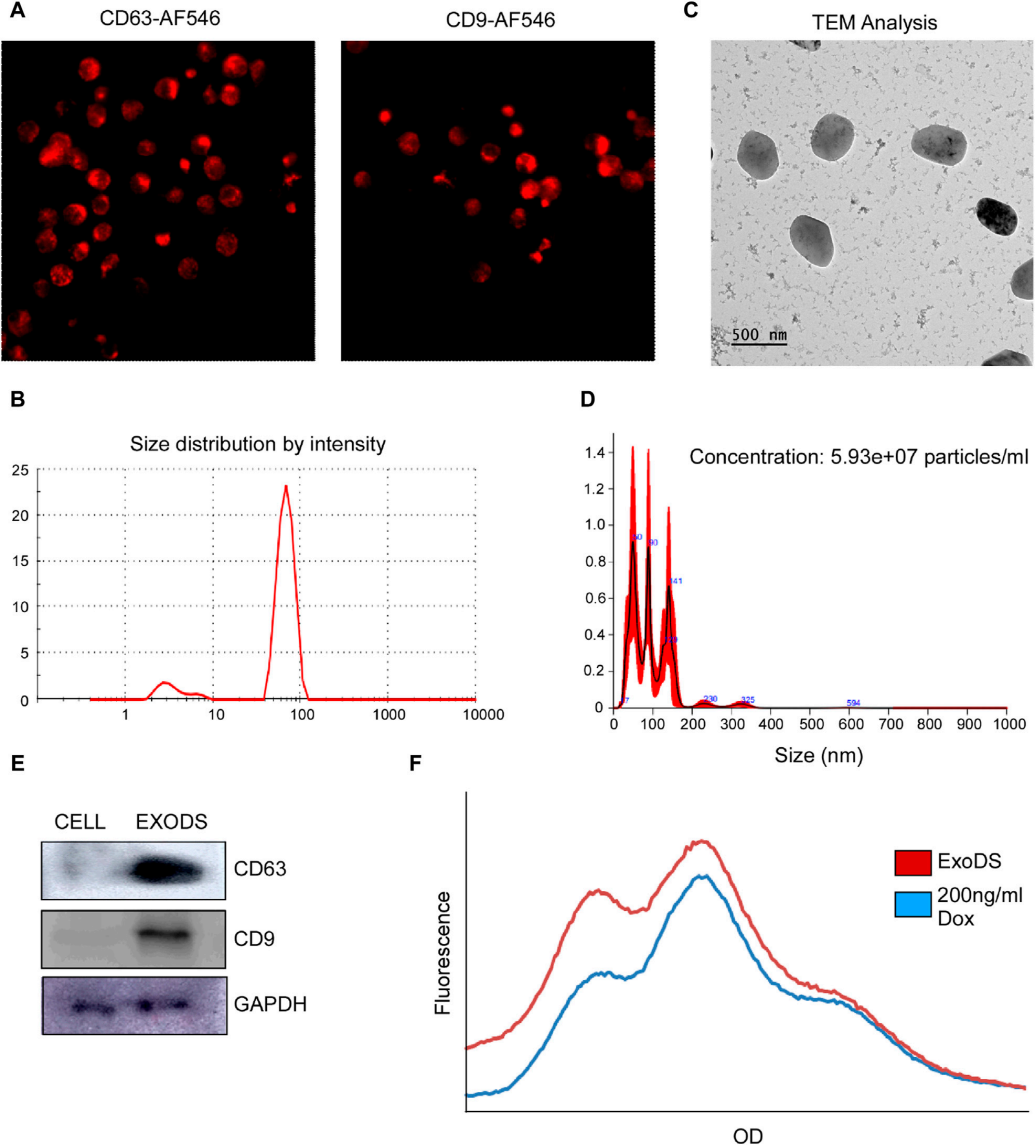
**(A)** Confocal images confirming the expression of exosomal markers CD63 and CD9 in induced mDCs. **(B)** DLS analysis confirming the size of the isolated particles to be within 150 nm as exosomes. **(C)** TEM was carried out to determine the shape of the exosomes isolated. **(D)** NTA of the isolated exosomes was carried out to determine the size range and concentration of the particles. **(E)** Western blot analysis of CD63, CD9, and GAPDH of the isolated exosomes was carried out to confirm the molecular signature of exosomes. **(F)** Histogram plot showing the concentration of doxorubicin packaged within ExoDS to be approximately 200 ng/mL.

Next, we analyzed whether mDCs generated using DendroSURE™ Cell Culture Media secrete exosomes. To that end, we conducted an image analysis using CD63 and CD9 antibodies. Our results showed that mDCs produce exosomes after 48 h of culture with DendroSURE™ Cell Culture Media (Figure 2A). To load exosomes with doxorubicin, we cultured mDCs with DendroSURE™ and doxorubicin at a concentration of 1.8 μg/mL for 24 h. After 24 h, we harvested the condition media and isolated exosomes using the Exosure exosome isolation reagent. Following exosome isolation, we characterized the isolated exosomes. We first conducted DLS analysis (Figure 2B) and AFM analysis (Supplementary Figure S1) of the exosome to identify the size range of the isolated particles. To understand the shape of the isolated exosomes, we conducted a transmission electron microscopy (TEM) analysis (Figure 2C). We also conducted NTA to understand the number of particles isolated (Figure 2D). Lastly, to verify the molecular nature of the isolated exosomes, we analyzed key endosomal markers, including CD63 and CD9 (Figure 2E). To analyze the concentration of doxorubicin packaged with exosomes, we conducted fluorimetry (Figure 2F) and HPLC analysis (see Supplementary Material). Both methods confirmed that doxorubicin was packaged within mDC-derived exosomes, and the concentration was 0.2 ng/µL, showing ∼11% packaging efficiency. Since we used an endogenous drug-loading approach, where the mDCs were directly incubated with doxorubicin, we also analyzed whether mDCs incubated with doxorubicin showed any apoptosis following treatment with 1.8 µg/mL of doxorubicin for 24 h. To understand the same, we conducted an Annexin V assay and a Western blot analysis. Supplementary Figure S2 shows that mDCs incubated with doxorubicin for 24 h showed an ∼1.9-fold increase in Annexin V concentration (left panel); furthermore, the expression of caspase-8 and LC3 increased in mDCs after doxorubicin treatment (right panel).

### ExoDS showed efficient targeting of cancer cells and cancer stem cells

**FIGURE 3:**
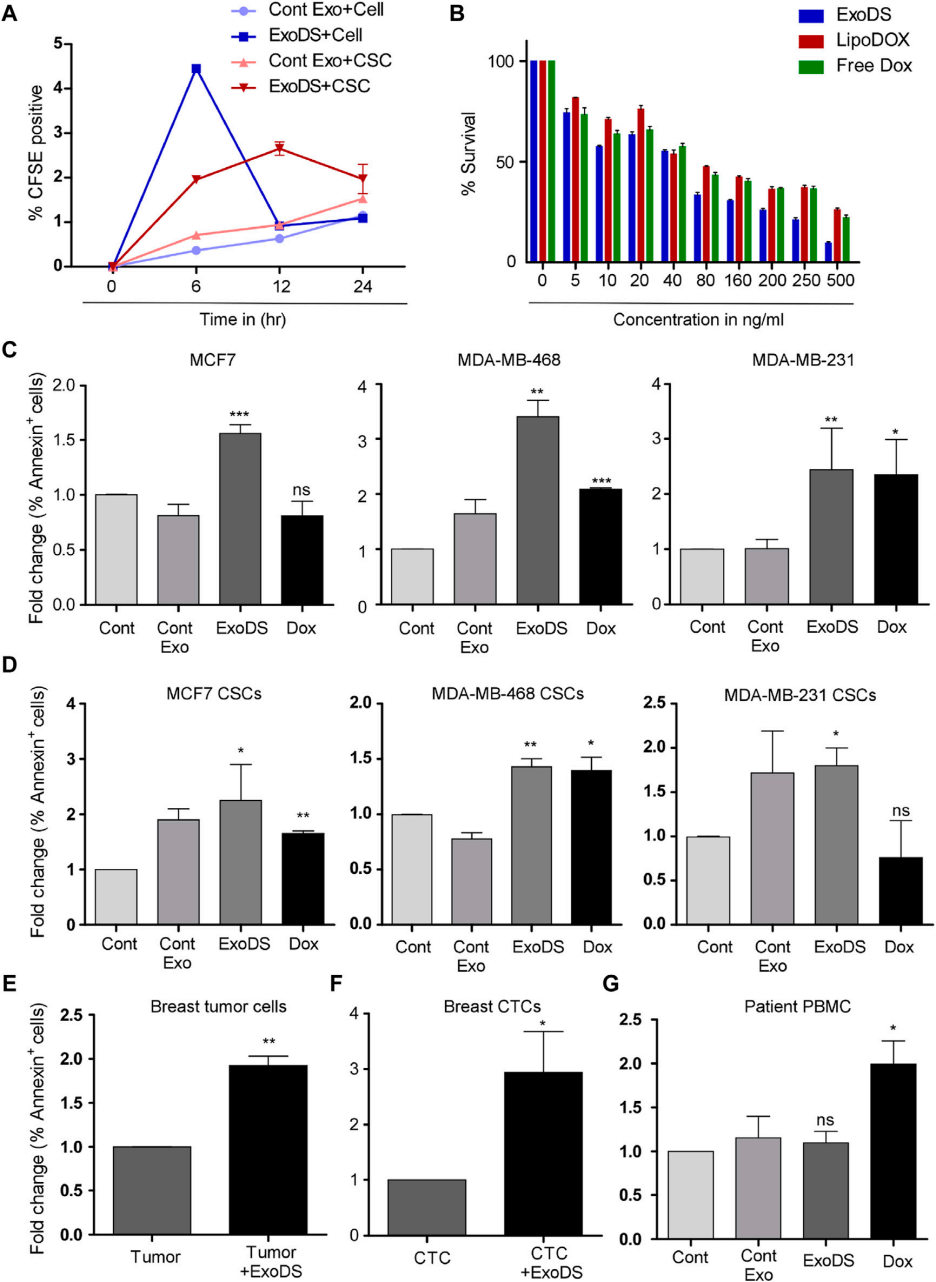
**(A)** Flow cytometric analysis of the internalization of CFSE-tagged ExoDS within MDA-MB-231 cancer cells and cancer stem cells in a time-dependent manner. Data were pooled from three independent experiments and represented in graphical format. **(B)** MTT assay demonstrating the efficacy of ExoDS in targeting MDA-MB-231 cancer cells compared to Lipo-Dox and free Dox. Data are represented as the average percentage pooled from three independent experiments. **(C)** Bar graph showing the efficacy of ExoDS in targeting both hormone receptor-positive MCF7 cells and triple-negative MDA-MB-231 and MDA-MB-468 cancer cells. **(D)** Bar graph showing the efficacy of ExoDS in targeting cancer stem cells derived from both hormone receptor-positive MCF7 cells and triple-negative MDA-MB-231 and MDA-MB-468 cancer cells. **(E)** Flow cytometric analysis of the percentage of Annexin V-positive cells before and after the treatment of breast tumor-derived cells with 5 ng/mL of ExoDS. Data from five independent experiments were pooled and represented in graphical format. **(F)** Flow cytometric analysis of the percentage of Annexin V-positive cells before and after the treatment of CTCs with 5 ng/mL of ExoDS. Data from six independent experiments were pooled and represented in graphical format. **(G)** Bar graph representing the percentage of apoptosis in PBMCs isolated from breast cancer patients following treatment with ExoDS and free Dox. Data were pooled from four independent experiments. The error bar indicates SEM; * indicates *p* < 0.05, ** indicates *p* < 0.01, and *** indicates *p* < 0.001.

To monitor the internalization potential of control exosomes derived from THP1 cells and ExoDS, we tagged THP1 cells and mature dendritic cells with the live cell-tagging CFSE dye. This led to the secretion of CFSE-tagged exosomes. ExoDS tagged with CFSE dye showed that it can be internalized significantly into cancer cells at 6 h (*p* < 0.0001) and cancer stem cells at 6 h (*p* < 0.0001) compared to control exosomes derived from the THP1 cell line, which enter at a much later time point of ∼12 h–24 h (Figure 3A). Next, to evaluate the cytotoxic effect of ExoDS on cancer cells, we conducted the MTT assay. We treated MDA-MB-231 cells with various concentrations of ExoDS, Lipo-Dox, and free doxorubicin for 48 h. Our results showed that ExoDS (IC_50_ = 74.28 ng) is significantly more effective than liposomal doxorubicin (IC_50_ = 164.11 ng) (*p* < 0.05) and free doxorubicin (IC_50_ = 145.62 ng) (*p* < 0.05) in targeting cancer cells (Figure 3B). In order to understand the efficiency of ExoDS compared with the unconjugated form of doxorubicin in inducing apoptosis in cancer cells (Figure 3C) and cancer stem cells (Figure 3D) derived from MCF-7, MDA-MB-468, and MDA-MB-231 cells, we performed an apoptosis assay using Annexin V. Our analysis showed that ExoDS (5 ng/mL) induced almost comparable killing compared to the unconjugated form of doxorubicin (1.8 μg/mL) at an almost 400-fold lower concentration in MCF-7, MDA-MB-468, and MDA-MB-231-derived cancer cells and cancer stem cells. Furthermore, Ki-67 analysis also showed a significant (*p* < 0.05) decrease in the expression of Ki-67 following treatment with 5 ng/mL ExoDS compared to the same concentration of Lipo-Dox and free Dox (Supplementary Figure S3).

### ExoDS efficiently induced apoptosis in breast cancer tissue-derived tumor cells

To check the efficiency of ExoDS in targeting cancer cells directly isolated from breast tumor tissue, we isolated tumor cells from breast cancer tissue and cultured them. Of the 13 tissues collected from KIMS Hospital, Bhubaneswar, we established sterile and stable primary cultures from 5 tumor tissues. The primary culture established from the five tissue samples was used next to test the efficacy of ExoDS. To that end, we treated the cells with 5 ng/mL ExoDS for 48 h, following which the cells were harvested and stained with the Annexin V antibody. Flow cytometric analysis confirmed that ExoDS caused significant apoptosis in tumor cells compared to the untreated control (Figure 3E). We also tested ExoDS on CTCs isolated from patient blood. CTCs were isolated and cultured as previously described. Of the blood samples collected from 13 tumor patients, CTC culture was established in 6 samples. The primary cultures of CTCs were treated with 5 ng/mL ExoDS for 48 h, after which the cells were harvested and stained with the Annexin V antibody. Similar to our previous observation, we found that ExoDS caused significant apoptosis in CTCs compared to the untreated control (Figure 3F).

### ExoDS failed to induce apoptosis in healthy cells

To confirm whether ExoDS also induced apoptosis in healthy cells, we isolated and cultured PBMCs from the blood of healthy donors and breast cancer patients. Following the culture of PBMCs, we treated them with similar concentrations of ExoDS and free Dox. Our flow cytometric analysis of Annexin V-positive cells showed that free Dox induced significant apoptosis in PBMCs compared to the control, whereas ExoDS did not induce any significant apoptosis in PBMCs (Supplementary Figure S4; Figure 3G). Similar results were also observed in the immortalized HEK293 cells (Supplementary Figure S5). To further validate the effect of ExoDS on healthy cells, we isolated and cultured normal mammary cells from tumor-adjacent normal tissue. Of the six tumor-adjacent normal tissue samples that we received, a successful culture was established from three tissues. These cells were treated with 5 ng/mL ExoDS for 48 h, and the percentage of Annexin V-positive cells was observed by flow cytometry (Supplementary Figure S6). Data suggested that ExoDS failed to induce apoptosis in healthy cells. This proved that ExoDS is specific toward tumor cells and not healthy cells.

### ExoDS showed a potent tumoricidal effect *in vivo*

To validate the effect of ExoDS *in vivo*, we induced triple-negative breast tumors in NOD/SCID mice using MDA-MB-231 cells. Following tumor development, we randomized the mice into four groups, each having two mice: Dox, ExoDS, cont exo (THP1-derived exosomes), and saline. The Dox group was injected intravenously with 20 ng of Dox in 100 µL of 1× sterile-filtered PBS, and the ExoDS group was injected with the 20 ng equivalent of Dox in 100 µL of ExoDS solution. The cont exo group was injected with 100 µL of the cont exo solution. The treatment regimen continued for 4 weeks, with doses given at 48-h intervals.

**FIGURE 4:**
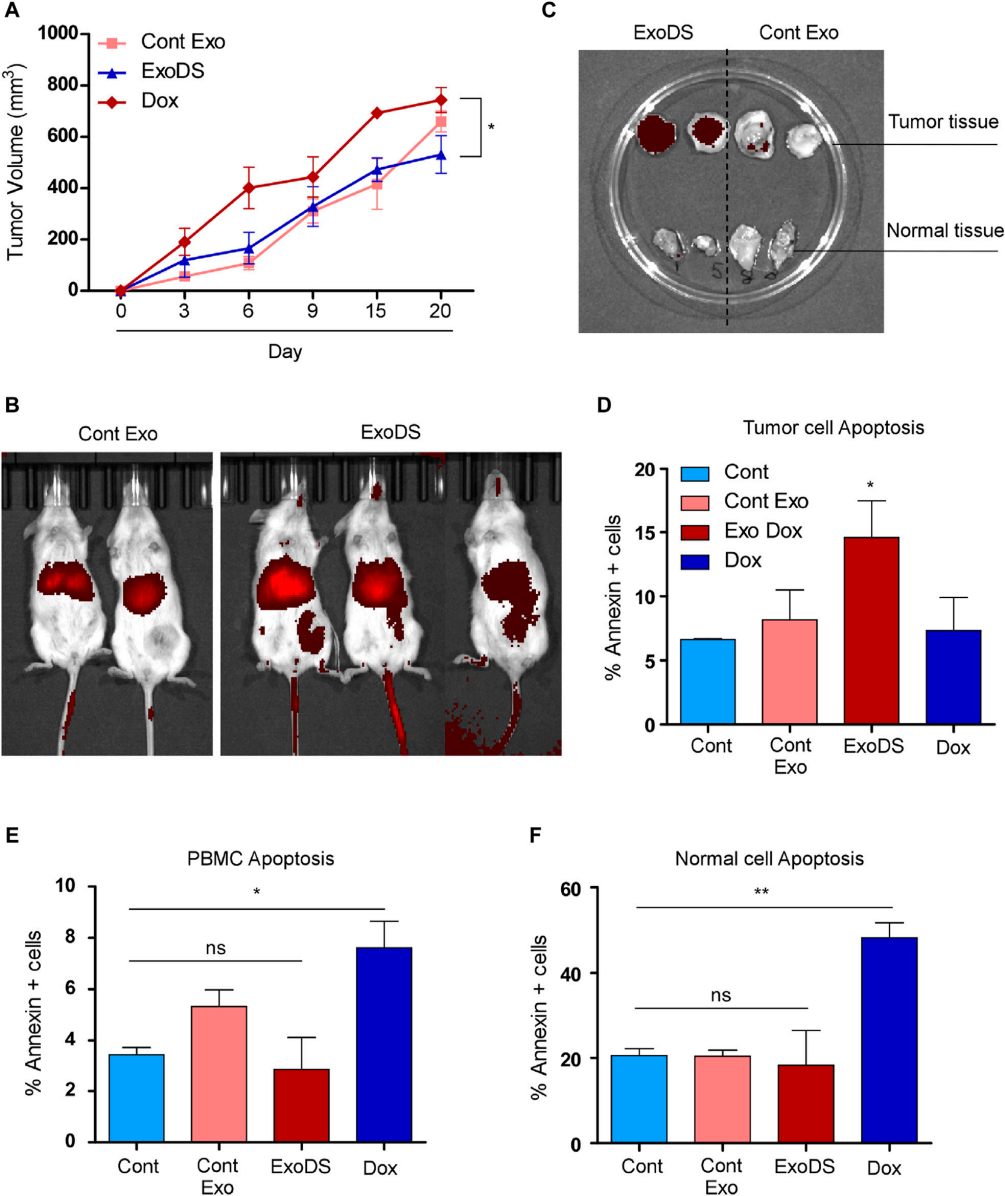
**(A)** Line graph showing the tumor volume of mice as a function of time. **(B)** Whole-body imaging of NOD/SCID mice showing the localization of the DiR-tagged exosomes (control exosomes and ExoDS). *In vivo* image confirmed that ExoDS specifically localized within the tumor tissue and not the control exosomes. **(C)** Images of excised tumors showing the specific localization of ExoDS only in the tumor tissue and not in the normal mammary tissue. **(D)** Bar graph showing the percentage of Annexin V-positive cells following the treatment of mice bearing tumor with saline (Cont), cont exo, ExoDS, and free Dox. **(E)** Bar graph showing the percentage of Annexin V-positive cells in PBMCs isolated from the tumor-bearing mice treated with saline (Cont), cont exo, ExoDS, and free Dox. **(F)** Bar graph showing the percentage of Annexin V-positive cells in normal mammary cells isolated from the tumor-bearing mice treated with saline (Cont), cont exo, ExoDS, and free Dox. Data were pooled from two independent biological replicates. The error bar indicates SEM; * indicates *p* < 0.05, ** indicates *p* < 0.01, and *** indicates *p* < 0.001.

We monitored the weight of each mouse and analyzed the tumor volume (in mm^3^) on days 1, 3, 6, 9, 15, and 20 (Figure 4A). Figure 4A shows a significant reduction in the increase in tumor volume following treatment with ExoDS compared to treatment with the unconjugated doxorubicin control. To confirm the distribution of ExoDS *in vivo*, whether it is targeted toward the tumor site or not, we intravenously injected DiR-tagged ExoDS to track their distribution across the entire body of the mouse. Figure 4B shows that our ExoDS is specifically targeted toward the tumor site at the right mammary fat pad, and it was localized into the tumor site within 12 h of injection. Furthermore, following the completion of the treatment regimen, we analyzed the DiR dye intensity in the tumor mass and the mammary fat pad tissue in the left mammary pad, where no tumor was induced. Figure 4C shows the localization of ExoDS specifically in the tumor site and not in the adjacent normal mammary fat pad tissue. We performed the Annexin V assay after extracting cells from the tumor mass (right mammary fat pad), healthy cells from the left mammary fat pad, and PBMCs isolated from mouse blood. Figure 4D shows that our ExoDS can induce significant killing in the tumor mass when compared to unconjugated doxorubicin at an equivalent concentration of 20 ng in 100 µL of 1× PBS. Furthermore, higher expression of the apoptotic marker caspase-8 and the autophagy marker LC3 was observed in ExoDS-treated tumor tissue by Western blot analysis (Supplementary Figure S7). Interestingly, qPCR analysis of stemness markers *Nanog*, *Oct4*, and *Sox2* in ExoDS-treated tumor tissue revealed a significant reduction in the expression of stemness markers *Nanog* and *OCT4* following treatment with ExoDS compared to free Dox (Supplementary Figure S7). Figure 4E shows that ExoDS has no toxic effect on the PBMCs isolated from the whole blood of mice in both groups when compared to unconjugated doxorubicin at an equivalent concentration of 20 ng in 100 µL of 1× PBS. Figure 4F shows that our ExoDS has no toxic effect on the cells isolated from the adjacent mammary fat pad without any tumor and induces no killing in the healthy mammary fat pad tissue when compared to unconjugated doxorubicin at an equivalent concentration of 20 ng in 100 µL of 1× PBS.

## Discussion

Multiple research studies have unveiled the potential of nanovesicles “exosomes” as carriers of small molecules or drugs for the treatment of a plethora of diseases both *in vitro* and *in vivo* (Hadla et al., 2016). Reports showed the importance of drug-loaded exosomes for clinical usage to treat patients and for diagnostic purposes (Kim et al., 2016). In our study, we highlighted the most cardiotoxic–chemotherapy drug “doxorubicin” as a drug cargo within mature dendritic cell-derived exosomes. mDC-derived exosomes were selectively used to target tumor cells and relapse-causing cancer stem cells. Our results demonstrated that ExoDS can induce comparable apoptosis in breast cancer cells and cancer stem cells, even at a 400-fold lower concentration when compared to free doxorubicin. ExoDS is not toxic toward healthy mammary cells or PBMCs derived from patient blood and healthy individuals. We further observed that ExoDS did not induce any significant apoptosis in the immortalized HEK293 cells, which might indicate the specificity of ExoDS toward breast cancer cells, although further validation is required. A similar study was conducted on primary tumor cells from biopsy samples of breast tumor tissue and normal cells isolated from adjacent normal mammary tissue. ExoDS showed no toxicity toward the PBMCs and normal mammary tissues, although it was specifically internalized by the tumor cells within 12 h of injection, confirming that ExoDS has no off-targeting effects on healthy cells. Our findings are similar to those observed by Zuo et al. (2022).

This targeting mechanism of ExoDS may be attributed to a variety of membrane receptors like integrin α and β chains (αMβ2), ICAM-1, MFG-E8, TNF, FasL, CD86, CD80, CD40, CD83, MHC-I, MHC-II, NKG2D, IL-15Rα, CD21, CD11c, and CCR7, which help mDC-derived exosomes identify cancer cells. Evidence supports that ICAM1 binds to MUC1, which is known to be overexpressed in cancer cells and cancer stem cells, and CD80 binds to CTLA4, which is an immunosuppressive marker that is also expressed in cancer stem cells. Moreover, CD80 binds to PD-L1, which is overexpressed up to 3-fold in breast CSCs. NKG2D ligands are often overexpressed in cancer and infected cells but are rarely expressed in normal tissues. The expression of NKG2DLs on the surface of tumor cells is induced by transcriptional upregulation due to cellular or genomic stress, and while it is usually expressed in most epithelial-derived tumor cells, it is rarely detected in healthy adult tissues (Munich et al., 2012; Luo et al., 2023). Human mDCs have the ability to kill tumor cells via TRAIL, FasL expression, or TNF-α, perforin, and granzyme secretion. Furthermore, we found that the endogenous packaging method of doxorubicin within ExoDS increased the expression of apoptotic markers like caspase-8 and Annexin V ∼1.9-fold within mDCs. Kowal et al. demonstrated the presence of caspase-8 within human mature dendritic cell-derived exosomes (Kowal et al., 2016). Since exosomal content mirrors the parent cell content, these mDC-derived tumoricidal and apoptotic agents may be loaded with ExoDS, which might potentiate their ability to target tumor cells. Further research is still warranted to decipher the complete proteome of ExoDS that helps in targeting cancer cells and cancer stem cells.

In our study, we restricted drug loading using the endogenous loading method only. Although this method yielded a lower concentration of doxorubicin loading in mDC-derived tumor-specific exosomes, it maintained the natural integrity of the exosomal membrane and also ensured replicability in drug loading in each batch. The integrity of the exosomal membrane further accelerated the initial adhesion to the tumor-specific cells and cancer stem cells as cellular adhesion proteins like integrins, lectins, and proteoglycans were intact, which also contributed to rapid cellular uptake by the recipient cells. Various research groups have demonstrated that packaged Dox maintains a favorable intracellular concentration for a prolonged period of time and is also not subjected to multidrug resistance transporter-mediated efflux (Kapse-Mistry et al., 2014; Liu et al., 2021). Thus, ExoDS can potentially internalize into relapse-causing cancer stem cells, which are characterized by the enhanced presence of MDR efflux pumps, and kill them efficiently, unlike conventional chemotherapeutics. Additionally, previous reports have corroborated our claim and showed that exosome-loaded doxorubicin, unlike free doxorubicin, is incapable of crossing the myocardial endothelium, which prevents doxorubicin deposition in the cardiac tissue and, hence, prevents cardiotoxicity (Alvarez-Erviti et al., 2011; György et al., 2015; Schindler et al., 2019).

Exosome-based cell-free therapy has additional advantages compared to cell therapy. Exosomes remain viable for longer and are easier to produce, store, and transport than bioengineered cells. They lack the risks associated with *in vivo* replication, which is plausible with cellular or viral therapies. Exosomes are also more resistant to immunomodulatory events *in vivo* (Pitt et al., 2016). Therefore, exosome-based cell-free therapy is rapidly emerging as the next generation of cancer therapy (Dutta, 2021; Dutta and Paul, 2022; Luo et al., 2023).

Furthermore, reports suggest that ExoDS is better than other existing drug delivery systems, such as nanoparticles or liposomal delivery methods. Exosomes are often considered the natural version of lipid nanoparticles. They have a unique composition like integrins, tetraspanins, and proteoglycans that may contribute to their excellent biocompatibility, stability in circulation, targeting specificity in cellular uptake, and their ability to pass through biological barriers. Exosomes also have lower toxicity than synthetic nanoparticles like liposomes. Additionally, due to the pharmacokinetics of liposomes in circulation, drugs can end up sequestered in the organs of the mononuclear phagocyte system, affecting liver and spleen function. Other nanoparticles, like carbon-based nanoparticles, can cause neurotoxicity, hepatotoxicity, pulmonary toxicity, immune toxicity, and cardiovascular toxicity. Superparamagnetic iron oxide nanoparticles (SPIONs) have emerged as a potential drug delivery system to treat cancer and other diseases. However, concerns related to the toxicity of SPIONs have limited their use until their properties are enhanced. On the other hand, ExoDS is produced from mDCs without any gene editing. This ensures high scale-up and reduced batch-to-batch variation (Inglut et al., 2020; Sharma et al., 2021; Liu et al., 2022; Al-Jipouri et al., 2023). Although much progress has been achieved, the development of therapeutic exosomes will require further refinements in exosome isolation techniques and the development of GMP-grade manufacturing of therapeutic exosomes (Dutta and Paul, 2022).

Overall, our findings reconfirmed that the natural tropism of mDC-derived exosomes and the ability to engineer them for enhanced target-specific selectivity and repurpose them as carriers for therapeutic cargo could create a new window in the treatment of breast cancer with an improved therapeutic index and survival outcomes in cancer patients. Furthermore, our study also, for the first time, confirmed the ability of mDC-derived exosomes to target CSCs. The pharmacokinetic and pharmacodynamic properties of ExoDS should be further studied to better understand the clinical potential of ExoDS.

## Data Availability

The original contributions presented in the study are included in the article/Supplementary Material; further inquiries can be directed to the corresponding authors.
