# Supplementary material for: ExoDS: a versatile exosome-based drug delivery platform to target cancer cells and cancer stem cells
Source: Front Bioeng Biotechnol. 2024 Jun 5;12:1362681. doi: 10.3389/fbioe.2024.1362681 (PMC11188490; doi:10.3389/fbioe.2024.1362681)
Supplement: Supplementary file 1 [file DataSheet1.docx]

**ExoDS:AVersatile Exosome-based Drug Delivery Platform to Target Cancer Cells and Cancer Stem Cells**

**Swastika Paul^1^, Shrikrishna Bhagat^1^, Lipsa Dash^1^, Himadri Das Mohapatra^1^, Sarita Jena^2^, Suresh K. Verma^3^*, Abhishek Dutta^1^***

Resource Table:

| **Reagent** | **Catalog Number** |
| --- | --- |
| CD14 | IM2580U (APC) |
| CD40 | B30636 (APC) |
| CD83 | IM2410U (FITC) |
| CD86 | B30647 (PC5.5) |
| Ki67 | 151212 (FIT C) |
| Annexin V | 640919 (APC) |
| LC3 | A5618 (ABclonal) |
| Caspase8 | A0215 (ABclonal) |
| CD9 | A19027 (ABclonal) |
| CD63 | A19023 (ABclonal) |
| GAPDH | A19056 (ABclonal) |
| β-actin | AC026 (ABclonal) |
| Hydrocortisone | CAS- No : 50-23-7, SLCF8402, Sigma- Aldrich |
| EGF | 585506 (Biolegend) |
| Insulin | SLCN1677 (Thermo) |
| FBS | 16000044 FBS, batch :2634126P, Thermo |
| B27 | GIBCO |
| Amph B | A011-20ML (HIMEDIA) |
| Penstrap | A018-100ML (HIMEDIA) |
| RPMI-1640 | AL028A-500ML (HIMEDIA) |
| DiR Dye | D12731 (Invitrogen) |
| Liposomal Doxorubicin | M.L.: G/28/396, SUN Pharmaceutical ind.ltd. |
| Doxorubicin | Lot. 2BDXD-JQ, TCI, Japan |
| MTT | 926640 (CDH) |
| HRP Goat Anti rabbit | AS014 (ABclonal) |


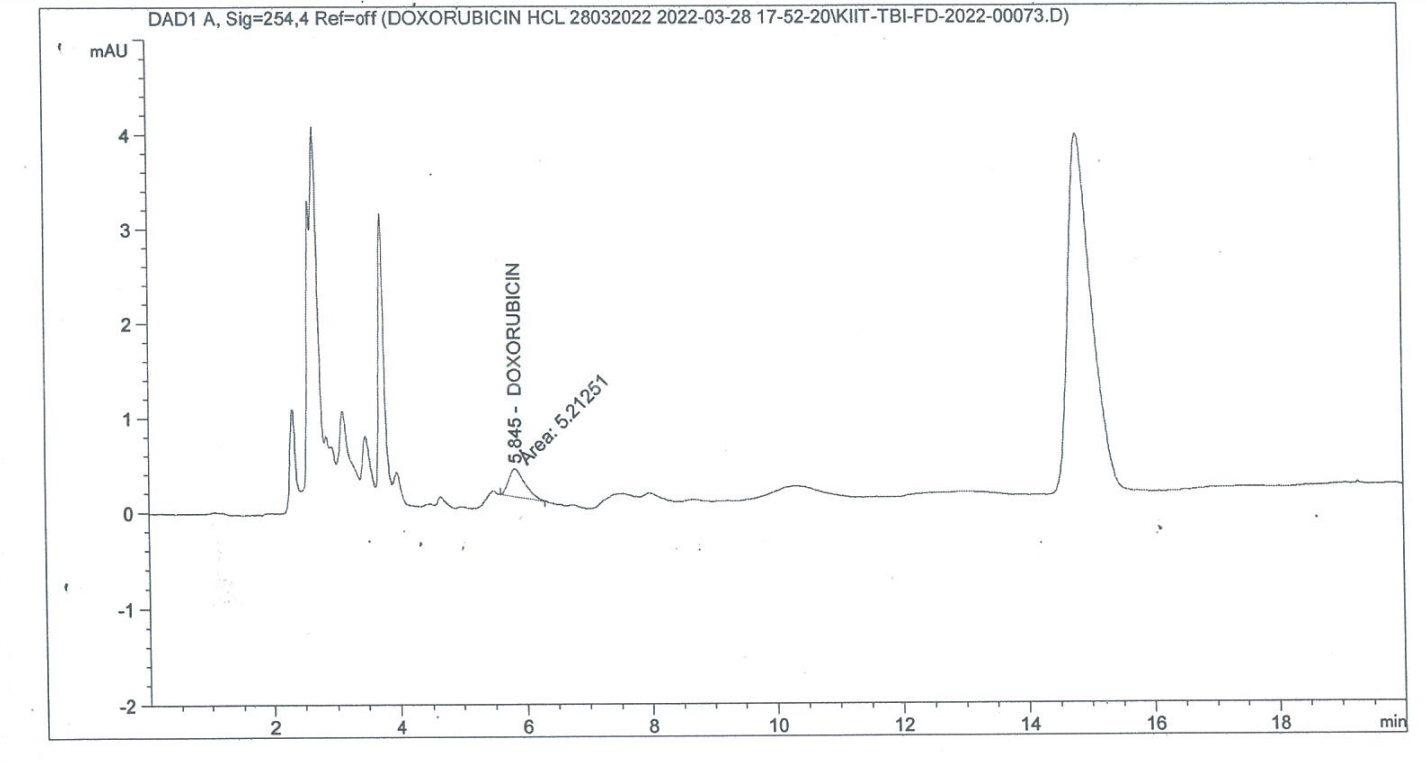


HPLC Chromatogram of ExoDS


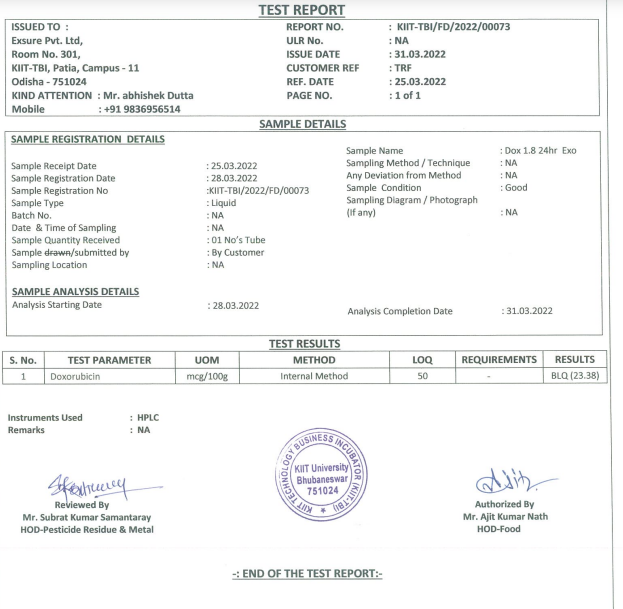


HPLC test report of ExoDS

Figure S1


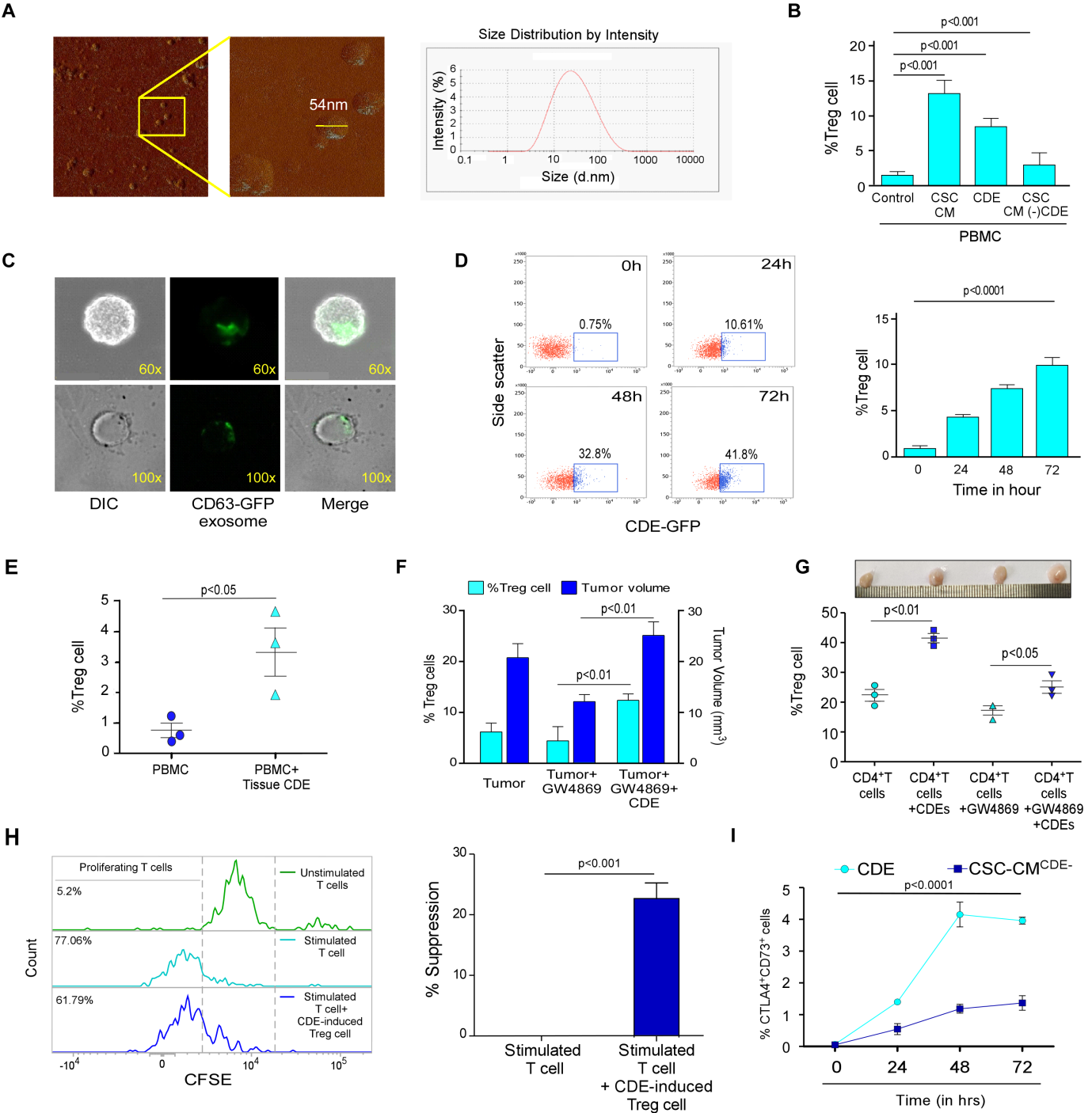


Figure S1- AFM imaging showing uniform size ExoDS.


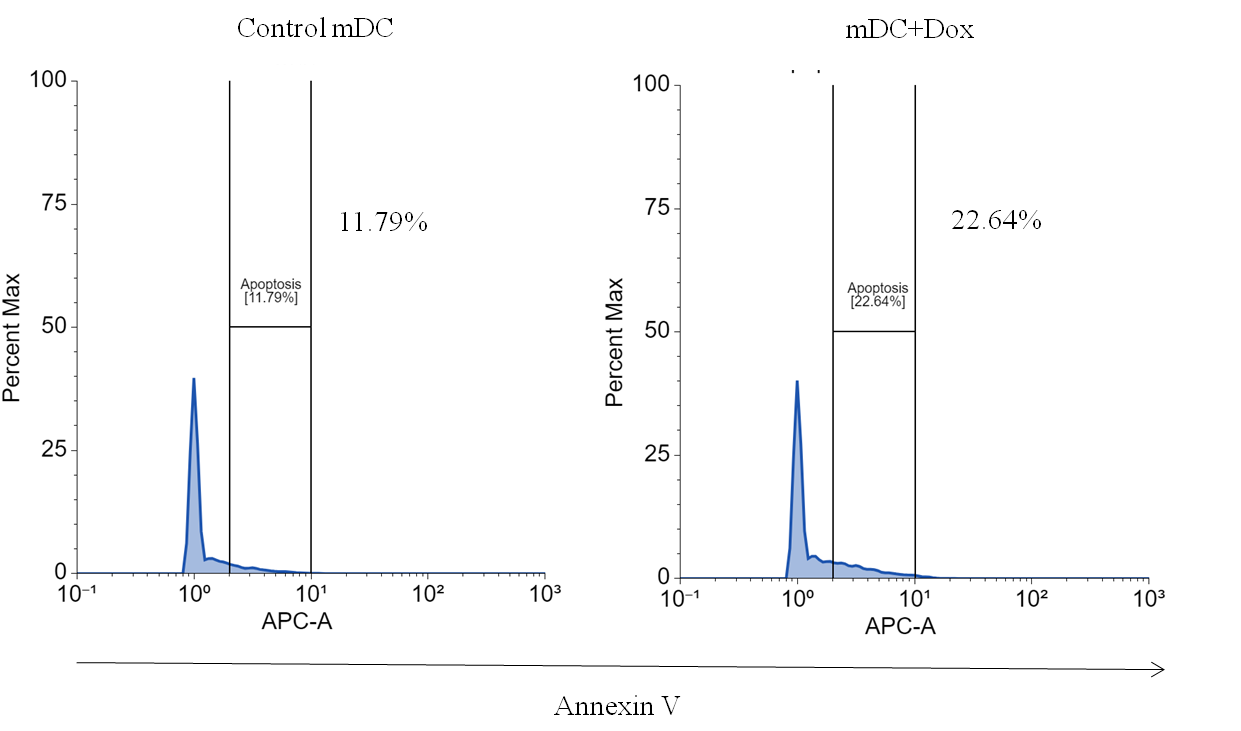
Figure S2

A


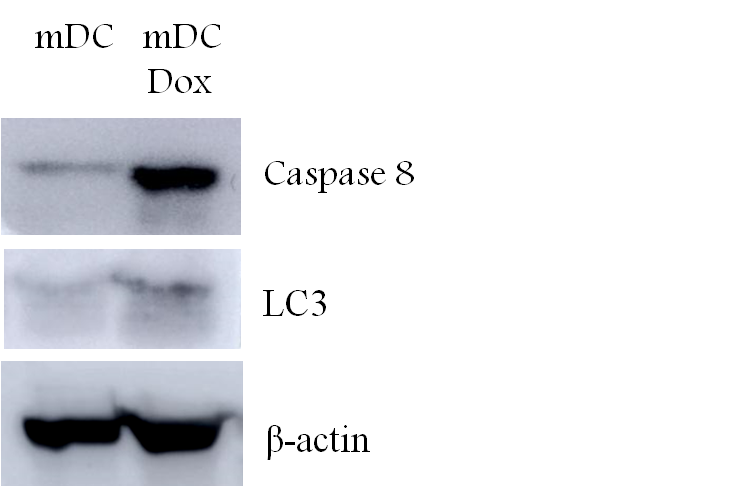


C


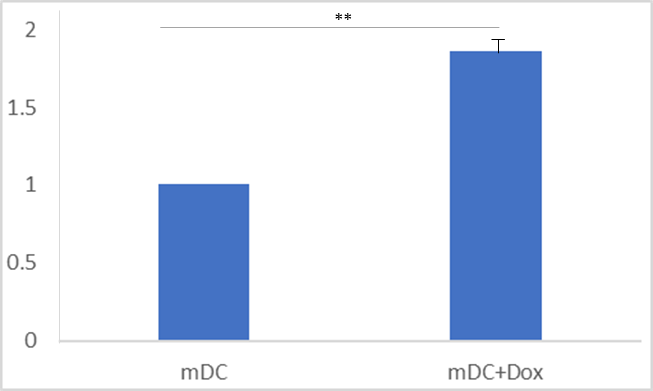
B

Figure S2- A: Flow cytometric analysis showing percentage Annexin V positive cells after treatment of mDCs with Doxorubicin (1.8μg/ml) for 24hrs. B: Bar graph representing the fold change of Annexin V expression in mDCs following treatment with doxorubicin (n=2). C: Western blot analysis showing expression of Caspase 8, and LC3 after treatment of mDCs with 1.8μg/ml Doxorubicin for 24hrs.

Figure S3


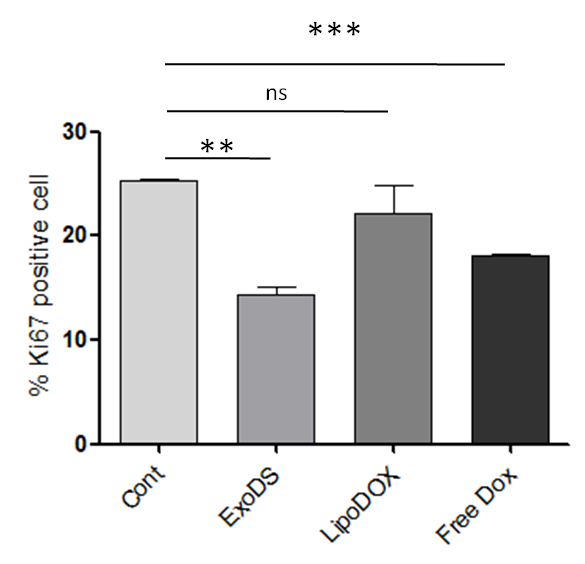


Figure S3-Flow cytometric analysis showing decrease in expression of Ki67 following treatment of MDA-MB-231 cells with 5ng/ml of ExoDS and free Dox as compared to Lipo Dox. Data was pooled from 3 independent experiments. The error bar indicates S.E.M.; * indicates p < .05; ** indicates p < .01, *** indicates p < .001, ns indicates non-significant.

Figure S4


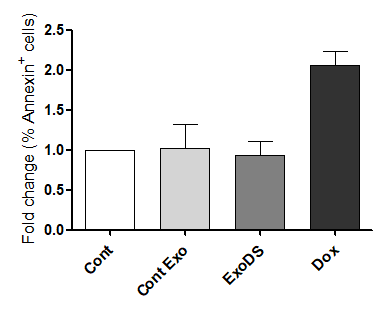


**

Figure S4- Bar graph representing the fold change in the % percentage Annexin V positive cells after treatment of PBMC isolated from healthy donor with Cont Exo, ExoDS and free dox. Data was pooled from 3 independent experiments. The error bar indicates S.E.M.

Figure S5


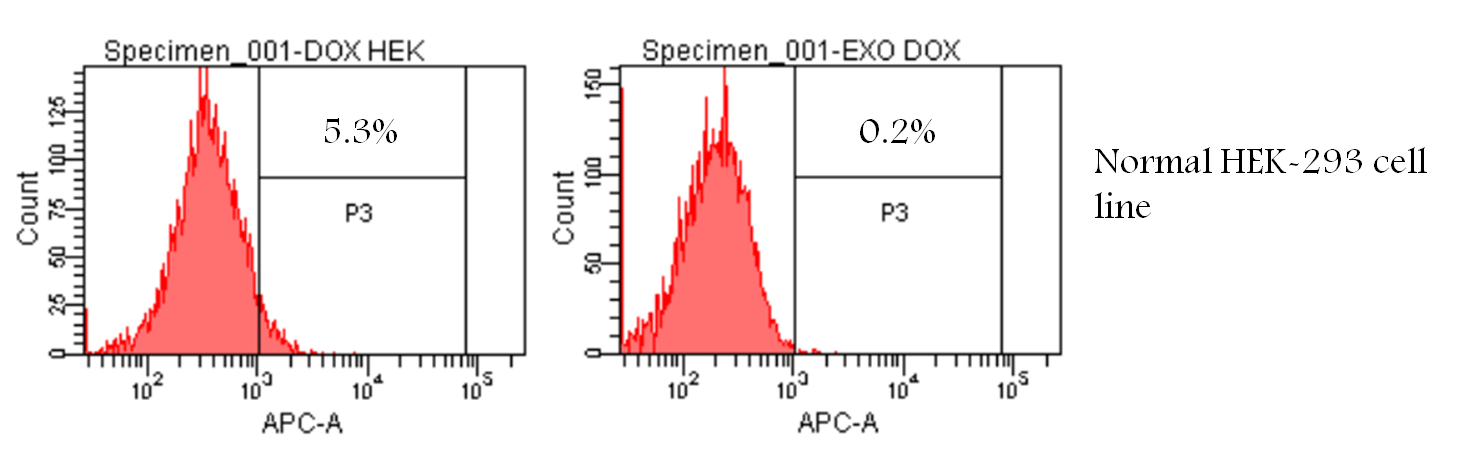


Figure S5- Histogram showing higher apoptosis in HEK293 cell line treated with free DOX as compared to ExoDS at same concentration of 5ng/ml


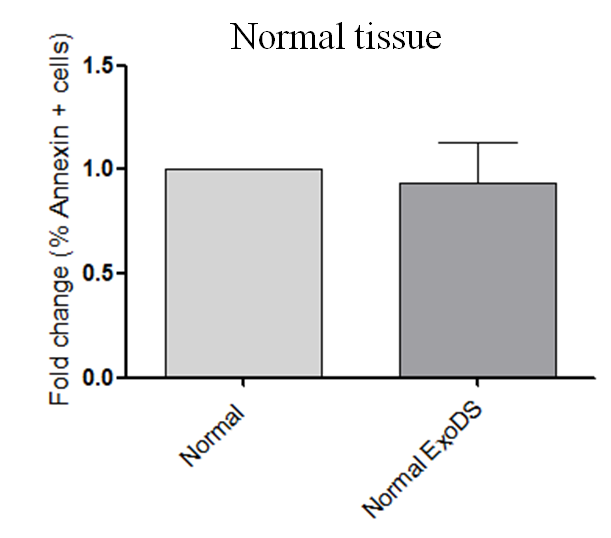

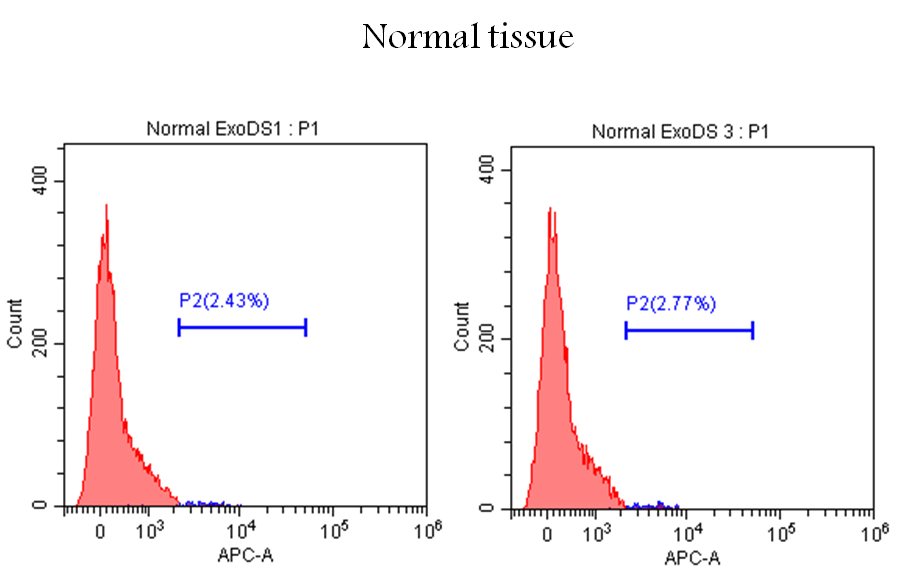
Figure S6

Figure S6- Flow cytometric analysis showing the inability of ExoDS in targeting normal mammary cells as no significant Annexin binding was observed following treatment in ExoDS. Bar graph representing data pooled from 3 independent biological replicate. The error bar indicates S.E.M.

Figure S7


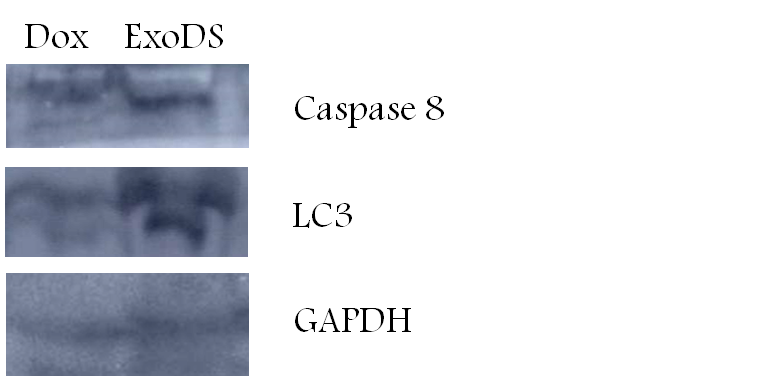


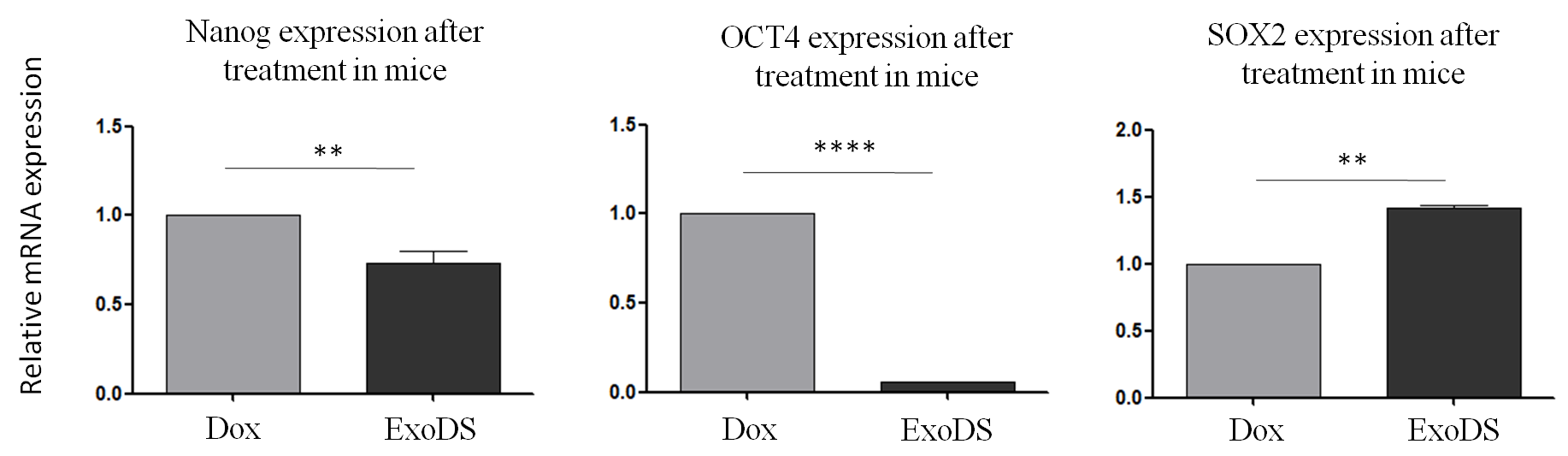


Figure S7- Upper panel: Western blot analysis showing higher expression of apoptotic marker Caspase 8 and autophagy marker LC3 in breast tumor tissue treated with ExoDS as compared to free Dox in murine xenograft model. Lower panel: Q-PCR analysis of stemness markers Nanog, OCT4, and SOX2 in breast tumor tissue treated with ExoDS and free Dox in murine xenograft model. The error bar indicates S.E.M.
